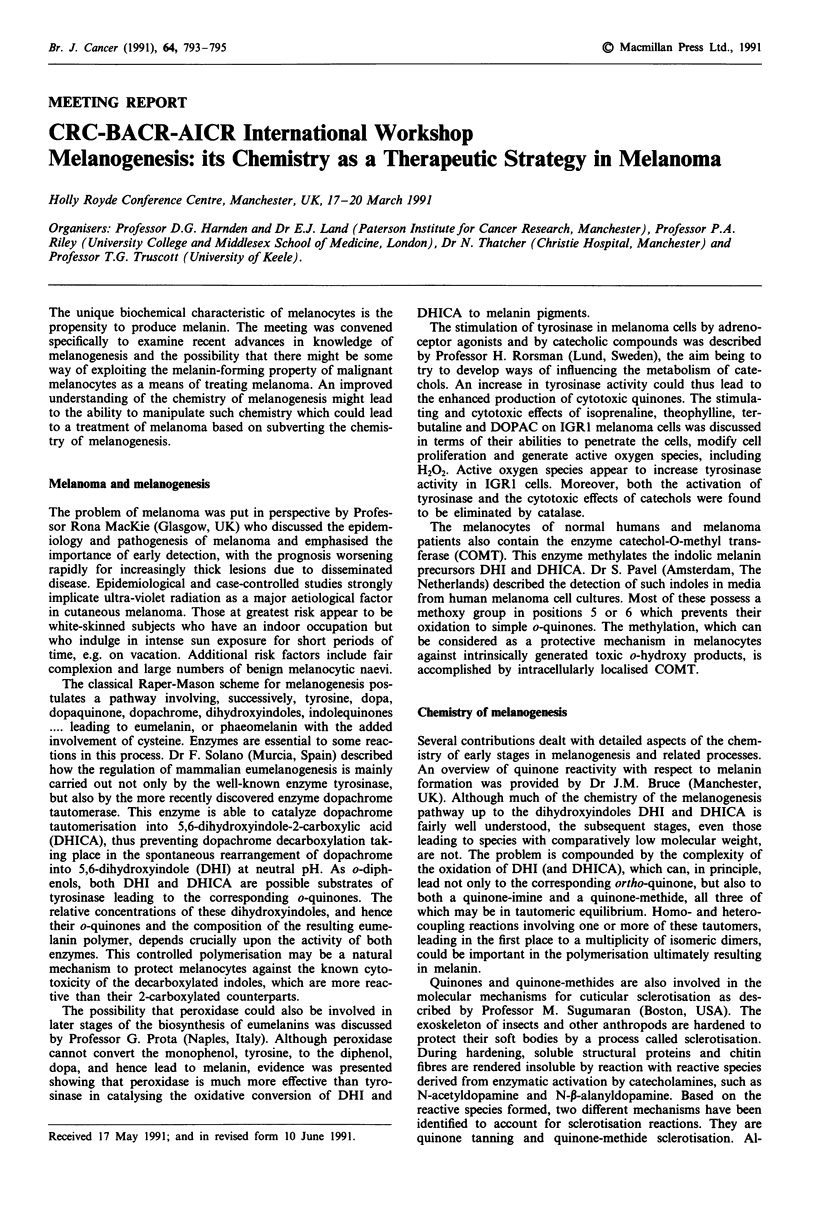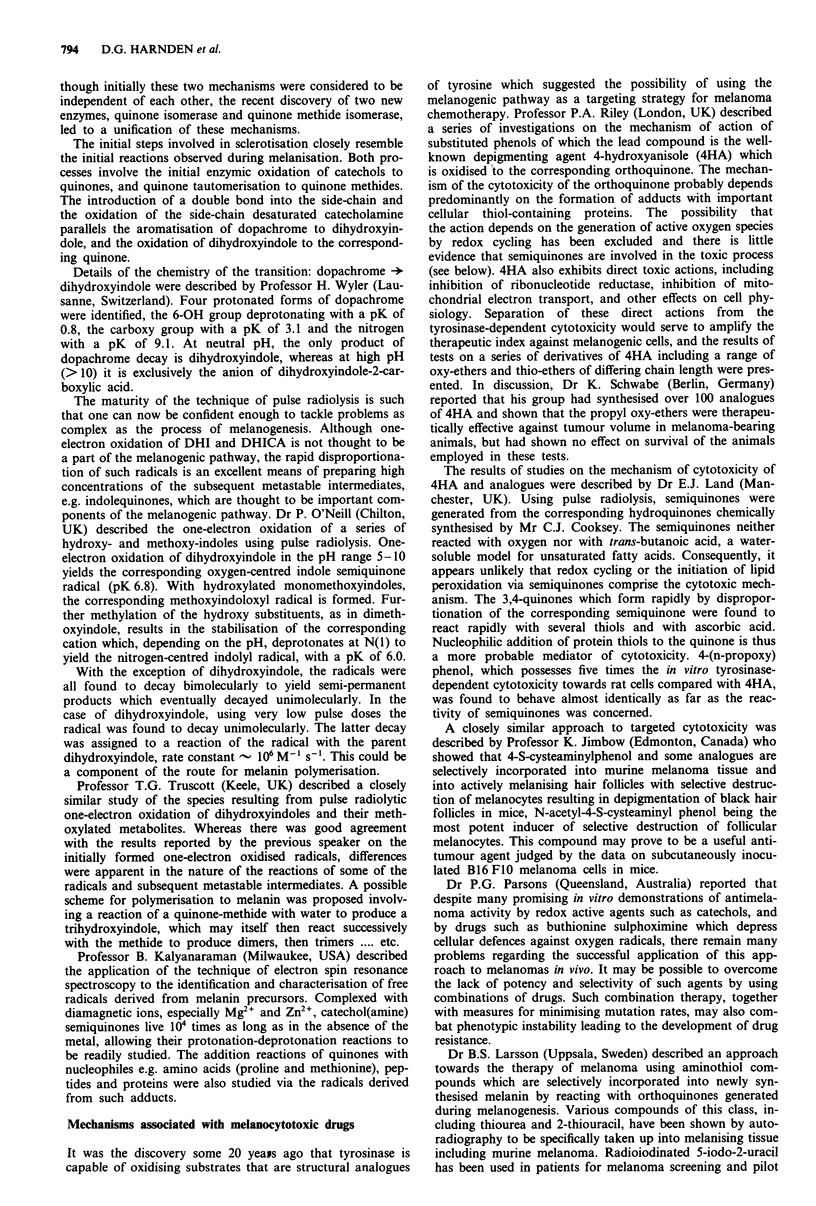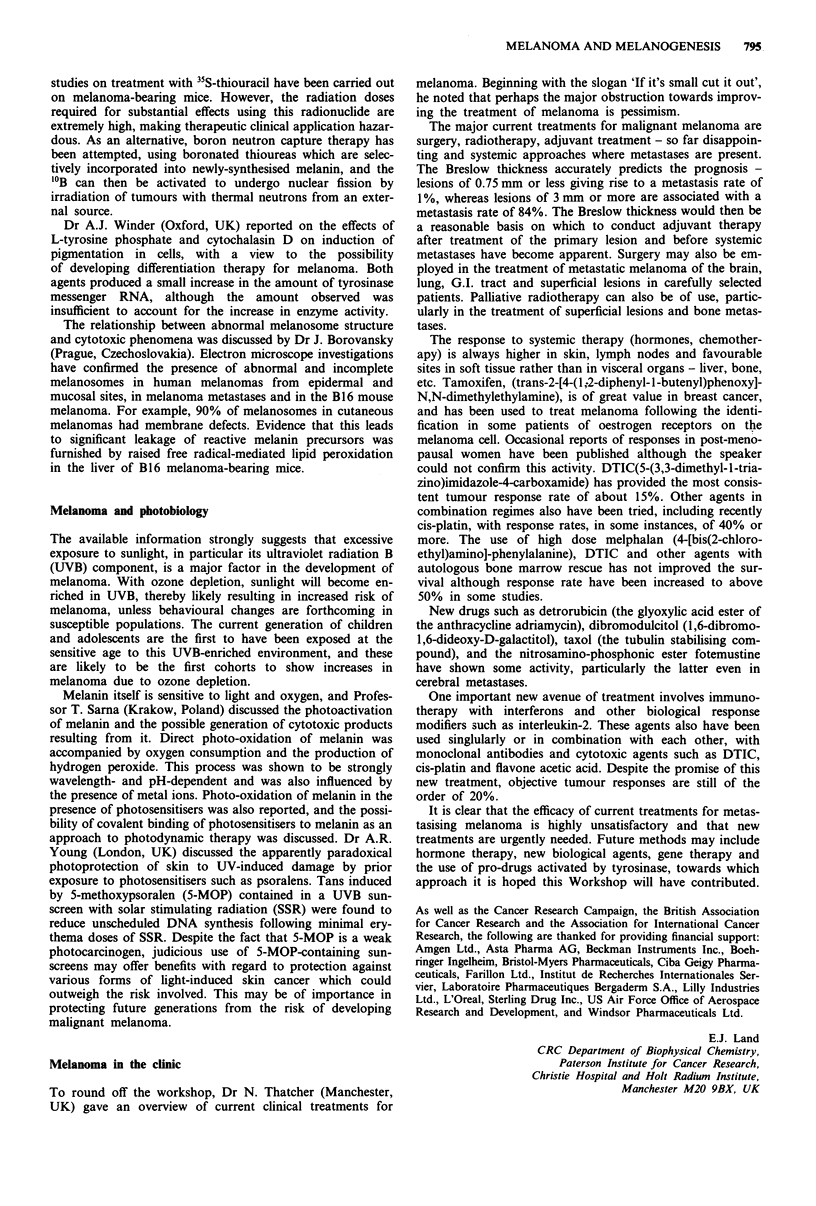# CRC-BACR-AICR International Workshop. Melanogenesis: its chemistry as a therapeutic strategy in melanoma.

**DOI:** 10.1038/bjc.1991.401

**Published:** 1991-10

**Authors:** E. J. Land

**Affiliations:** CRC Department of Biophysical Chemistry, Paterson Institute for Cancer Research, Christie Hospital and Holt Radium Institute, Manchester, UK.


Br. J. Cancer (1991), 64, 793–795 © Macmillan Press Ltd., 1991

MEETING REPORT

### CRC-BACR-AICR International Workshop Melanogenesis: its Chemistry as a Therapeutic Strategy in Melanoma

*Holly Royde Conference Centre, Manchester, UK, 17–20 March 1991*

*Organisers: Professor D.G. Harnden and Dr E.J. Land (Paterson Institute for Cancer Research, Manchester), Professor P.A. Riley (University College and Middlesex School of Medicine, London), Dr N. Thatcher (Christie Hospital, Manchester) and Professor T.G. Truscott (University of Keele).*

The unique biochemical characteristic of melanocytes is the propensity to produce melanin. The meeting was convened specifically to examine recent advances in knowledge of melanogenesis and the possibility that there might be some way of exploiting the melanin-forming property of malignant melanocytes as a means of treating melanoma. An improved understanding of the chemistry of melanogenesis might lead to the ability to manipulate such chemistry which could lead to a treatment of melanoma based on subverting the chemistry of melanogenesis.

### Melanoma and melanogenesis

The problem of melanoma was put in perspective by Professor Rona MacKie (Glasgow, UK) who discussed the epidemiology and pathogenesis of melanoma and emphasised the importance of early detection, with the prognosis worsening rapidly for increasingly thick lesions due to disseminated disease. Epidemiological and case-controlled studies strongly implicate ultra-violet radiation as a major aetiological factor in cutaneous melanoma. Those at greatest risk appear to be white-skinned subjects who have an indoor occupation but who indulge in intense sun exposure for short periods of time, e.g. on vacation. Additional risk factors include fair complexion and large numbers of benign melanocytic naevi.

The classical Raper-Mason scheme for melanogenesis postulates a pathway involving, successively, tyrosine, dopa, dopaquinone, dopachrome, dihydroxyindoles, indolequinones .... leading to eumelanin, or phaeomelanin with the added involvement of cysteine. Enzymes are essential to some reactions in this process. Dr F. Solano (Murcia, Spain) described how the regulation of mammalian eumelanogenesis is mainly carried out not only by the well-known enzyme tyrosinase, but also by the more recently discovered enzyme dopachrome tautomerase. This enzyme is able to catalyze dopachrome tautomerisation into 5,6-dihydroxyindole-2-carboxylic acid (DHICA), thus preventing dopachrome decarboxylation taking place in the spontaneous rearrangement of dopachrome into 5,6-dihydroxyindole (DHI) at neutral pH. As *o*-diphenols, both DHI and DHICA are possible substrates of tyrosinase leading to the corresponding *o*-quinones. The relative concentrations of these dihydroxyindoles, and hence their *o*-quinones and the composition of the resulting eumelanin polymer, depends crucially upon the activity of both enzymes. This controlled polymerisation may be a natural mechanism to protect melanocytes against the known cytotoxicity of the decarboxylated indoles, which are more reactive than their 2-carboxylated counterparts.

The possibility that peroxidase could also be involved in later stages of the biosynthesis of eumelanins was discussed by Professor G. Prota (Naples, Italy). Although peroxidase cannot convert the monophenol, tyrosine, to the diphenol, dopa, and hence lead to melanin, evidence was presented showing that peroxidase is much more effective than tyrosinase in catalysing the oxidative conversion of DHI and DHICA to melanin pigments.

The stimulation of tyrosinase in melanoma cells by adrenoceptor agonists and by catecholic compounds was described by Professor H. Rorsman (Lund, Sweden), the aim being to try to develop ways of influencing the metabolism of catechols. An increase in tyrosinase activity could thus lead to the enhanced production of cytotoxic quinones. The stimulating and cytotoxic effects of isoprenaline, theophylline, terbutaline and DOPAC on IGR1 melanoma cells was discussed in terms of their abilities to penetrate the cells, modify cell proliferation and generate active oxygen species, including $H_2O_2$. Active oxygen species appear to increase tyrosinase activity in IGR1 cells. Moreover, both the activation of tyrosinase and the cytotoxic effects of catechols were found to be eliminated by catalase.

The melanocytes of normal humans and melanoma patients also contain the enzyme catechol-O-methyl transferase (COMT). This enzyme methylates the indolic melanin precursors DHI and DHICA. Dr S. Pavel (Amsterdam, The Netherlands) described the detection of such indoles in media from human melanoma cell cultures. Most of these possess a methoxy group in positions 5 or 6 which prevents their oxidation to simple *o*-quinones. The methylation, which can be considered as a protective mechanism in melanocytes against intrinsically generated toxic *o*-hydroxy products, is accomplished by intracellularly localised COMT.

### Chemistry of melanogenesis

Several contributions dealt with detailed aspects of the chemistry of early stages in melanogenesis and related processes. An overview of quinone reactivity with respect to melanin formation was provided by Dr J.M. Bruce (Manchester, UK). Although much of the chemistry of the melanogenesis pathway up to the dihydroxyindoles DHI and DHICA is fairly well understood, the subsequent stages, even those leading to species with comparatively low molecular weight, are not. The problem is compounded by the complexity of the oxidation of DHI (and DHICA), which can, in principle, lead not only to the corresponding *ortho*-quinone, but also to both a quinone-imine and a quinone-methide, all three of which may be in tautomeric equilibrium. Homo- and hetero-coupling reactions involving one or more of these tautomers, leading in the first place to a multiplicity of isomeric dimers, could be important in the polymerisation ultimately resulting in melanin.

Quinones and quinone-methides are also involved in the molecular mechanisms for cuticular sclerotisation as described by Professor M. Sugumaran (Boston, USA). The exoskeleton of insects and other anthropods are hardened to protect their soft bodies by a process called sclerotisation. During hardening, soluble structural proteins and chitin fibres are rendered insoluble by reaction with reactive species derived from enzymatic activation by catecholamines, such as N-acetyldopamine and N-β-alanyldopamine. Based on the reactive species formed, two different mechanisms have been identified to account for sclerotisation reactions. They are quinone tanning and quinone-methide sclerotisation. Al-

Received 17 May 1991; and in revised form 10 June 1991.

though initially these two mechanisms were considered to be independent of each other, the recent discovery of two new enzymes, quinone isomerase and quinone methide isomerase, led to a unification of these mechanisms.

The initial steps involved in sclerotisation closely resemble the initial reactions observed during melanisation. Both processes involve the initial enzymic oxidation of catechols to quinones, and quinone tautomerisation to quinone methides. The introduction of a double bond into the side-chain and the oxidation of the side-chain desaturated catecholamine parallels the aromatisation of dopachrome to dihydroxyindole, and the oxidation of dihydroxyindole to the corresponding quinone.

Details of the chemistry of the transition: dopachrome → dihydroxyindole were described by Professor H. Wyler (Lausanne, Switzerland). Four protonated forms of dopachrome were identified, the 6-OH group deprotonating with a pK of 0.8, the carboxy group with a pK of 3.1 and the nitrogen with a pK of 9.1. At neutral pH, the only product of dopachrome decay is dihydroxyindole, whereas at high pH ($>10$) it is exclusively the anion of dihydroxyindole-2-carboxylic acid.

The maturity of the technique of pulse radiolysis is such that one can now be confident enough to tackle problems as complex as the process of melanogenesis. Although one-electron oxidation of DHI and DHICA is not thought to be a part of the melanogenic pathway, the rapid disproportionation of such radicals is an excellent means of preparing high concentrations of the subsequent metastable intermediates, e.g. indolequinones, which are thought to be important components of the melanogenic pathway. Dr P. O'Neill (Chilton, UK) described the one-electron oxidation of a series of hydroxy- and methoxy-indoles using pulse radiolysis. One-electron oxidation of dihydroxyindole in the pH range 5–10 yields the corresponding oxygen-centred indole semiquinone radical (pK 6.8). With hydroxylated monomethoxyindoles, the corresponding methoxyindoloxyl radical is formed. Further methylation of the hydroxy substituents, as in dimethoxyindole, results in the stabilisation of the corresponding cation which, depending on the pH, deprotonates at N(1) to yield the nitrogen-centred indolyl radical, with a pK of 6.0.

With the exception of dihydroxyindole, the radicals were all found to decay bimolecularly to yield semi-permanent products which eventually decayed unimolecularly. In the case of dihydroxyindole, using very low pulse doses the radical was found to decay unimolecularly. The latter decay was assigned to a reaction of the radical with the parent dihydroxyindole, rate constant $\sim 10^6 M^{-1} s^{-1}$. This could be a component of the route for melanin polymerisation.

Professor T.G. Truscott (Keele, UK) described a closely similar study of the species resulting from pulse radiolytic one-electron oxidation of dihydroxyindoles and their methoxylated metabolites. Whereas there was good agreement with the results reported by the previous speaker on the initially formed one-electron oxidised radicals, differences were apparent in the nature of the reactions of some of the radicals and subsequent metastable intermediates. A possible scheme for polymerisation to melanin was proposed involving a reaction of a quinone-methide with water to produce a trihydroxyindole, which may itself then react successively with the methide to produce dimers, then trimers .... etc.

Professor B. Kalyanaraman (Milwaukee, USA) described the application of the technique of electron spin resonance spectroscopy to the identification and characterisation of free radicals derived from melanin precursors. Complexed with diamagnetic ions, especially $Mg^{2+}$ and $Zn^{2+}$, catechol(amine) semiquinones live $10^4$ times as long as in the absence of the metal, allowing their protonation-deprotonation reactions to be readily studied. The addition reactions of quinones with nucleophiles e.g. amino acids (proline and methionine), peptides and proteins were also studied via the radicals derived from such adducts.

**Mechanisms associated with melanocytotoxic drugs**

It was the discovery some 20 years ago that tyrosinase is capable of oxidising substrates that are structural analogues of tyrosine which suggested the possibility of using the melanogenic pathway as a targeting strategy for melanoma chemotherapy. Professor P.A. Riley (London, UK) described a series of investigations on the mechanism of action of substituted phenols of which the lead compound is the well-known depigmenting agent 4-hydroxyanisole (4HA) which is oxidised to the corresponding orthoquinone. The mechanism of the cytotoxicity of the orthoquinone probably depends predominantly on the formation of adducts with important cellular thiol-containing proteins. The possibility that the action depends on the generation of active oxygen species by redox cycling has been excluded and there is little evidence that semiquinones are involved in the toxic process (see below). 4HA also exhibits direct toxic actions, including inhibition of ribonucleotide reductase, inhibition of mitochondrial electron transport, and other effects on cell physiology. Separation of these direct actions from the tyrosinase-dependent cytotoxicity would serve to amplify the therapeutic index against melanogenic cells, and the results of tests on a series of derivatives of 4HA including a range of oxy-ethers and thio-ethers of differing chain length were presented. In discussion, Dr K. Schwabe (Berlin, Germany) reported that his group had synthesised over 100 analogues of 4HA and shown that the propyl oxy-ethers were therapeutically effective against tumour volume in melanoma-bearing animals, but had shown no effect on survival of the animals employed in these tests.

The results of studies on the mechanism of cytotoxicity of 4HA and analogues were described by Dr E.J. Land (Manchester, UK). Using pulse radiolysis, semiquinones were generated from the corresponding hydroquinones chemically synthesised by Mr C.J. Cooksey. The semiquinones neither reacted with oxygen nor with *trans*-butanoic acid, a water-soluble model for unsaturated fatty acids. Consequently, it appears unlikely that redox cycling or the initiation of lipid peroxidation via semiquinones comprise the cytotoxic mechanism. The 3,4-quinones which form rapidly by disproportionation of the corresponding semiquinone were found to react rapidly with several thiols and with ascorbic acid. Nucleophilic addition of protein thiols to the quinone is thus a more probable mediator of cytotoxicity. 4-(n-propoxy) phenol, which possesses five times the *in vitro* tyrosinase-dependent cytotoxicity towards rat cells compared with 4HA, was found to behave almost identically as far as the reactivity of semiquinones was concerned.

A closely similar approach to targeted cytotoxicity was described by Professor K. Jimbow (Edmonton, Canada) who showed that 4-S-cysteaminylphenol and some analogues are selectively incorporated into murine melanoma tissue and into actively melanising hair follicles with selective destruction of melanocytes resulting in depigmentation of black hair follicles in mice, N-acetyl-4-S-cysteaminyl phenol being the most potent inducer of selective destruction of follicular melanocytes. This compound may prove to be a useful anti-tumour agent judged by the data on subcutaneously inoculated B16 F10 melanoma cells in mice.

Dr P.G. Parsons (Queensland, Australia) reported that despite many promising *in vitro* demonstrations of antimelanoma activity by redox active agents such as catechols, and by drugs such as buthionine sulphoximine which depress cellular defences against oxygen radicals, there remain many problems regarding the successful application of this approach to melanomas *in vivo*. It may be possible to overcome the lack of potency and selectivity of such agents by using combinations of drugs. Such combination therapy, together with measures for minimising mutation rates, may also combat phenotypic instability leading to the development of drug resistance.

Dr B.S. Larsson (Uppsala, Sweden) described an approach towards the therapy of melanoma using aminothiol compounds which are selectively incorporated into newly synthesised melanin by reacting with orthoquinones generated during melanogenesis. Various compounds of this class, including thiourea and 2-thiouracil, have been shown by autoradiography to be specifically taken up into melanising tissue including murine melanoma. Radioiodinated 5-iodo-2-uracil has been used in patients for melanoma screening and pilot studies on treatment with $^{35}$S-thiouracil have been carried out on melanoma-bearing mice. However, the radiation doses required for substantial effects using this radionuclide are extremely high, making therapeutic clinical application hazardous. As an alternative, boron neutron capture therapy has been attempted, using boronated thioureas which are selectively incorporated into newly-synthesised melanin, and the $^{10}$B can then be activated to undergo nuclear fission by irradiation of tumours with thermal neutrons from an external source.

Dr A.J. Winder (Oxford, UK) reported on the effects of L-tyrosine phosphate and cytochalasin D on induction of pigmentation in cells, with a view to the possibility of developing differentiation therapy for melanoma. Both agents produced a small increase in the amount of tyrosinase messenger RNA, although the amount observed was insufficient to account for the increase in enzyme activity.

The relationship between abnormal melanosome structure and cytotoxic phenomena was discussed by Dr J. Borovansky (Prague, Czechoslovakia). Electron microscope investigations have confirmed the presence of abnormal and incomplete melanosomes in human melanomas from epidermal and mucosal sites, in melanoma metastases and in the B16 mouse melanoma. For example, 90% of melanosomes in cutaneous melanomas had membrane defects. Evidence that this leads to significant leakage of reactive melanin precursors was furnished by raised free radical-mediated lipid peroxidation in the liver of B16 melanoma-bearing mice.

### Melanoma and photobiology

The available information strongly suggests that excessive exposure to sunlight, in particular its ultraviolet radiation B (UVB) component, is a major factor in the development of melanoma. With ozone depletion, sunlight will become enriched in UVB, thereby likely resulting in increased risk of melanoma, unless behavioural changes are forthcoming in susceptible populations. The current generation of children and adolescents are the first to have been exposed at the sensitive age to this UVB-enriched environment, and these are likely to be the first cohorts to show increases in melanoma due to ozone depletion.

Melanin itself is sensitive to light and oxygen, and Professor T. Sarna (Krakow, Poland) discussed the photoactivation of melanin and the possible generation of cytotoxic products resulting from it. Direct photo-oxidation of melanin was accompanied by oxygen consumption and the production of hydrogen peroxide. This process was shown to be strongly wavelength- and pH-dependent and was also influenced by the presence of metal ions. Photo-oxidation of melanin in the presence of photosensitisers was also reported, and the possibility of covalent binding of photosensitisers to melanin as an approach to photodynamic therapy was discussed. Dr A.R. Young (London, UK) discussed the apparently paradoxical photoprotection of skin to UV-induced damage by prior exposure to photosensitisers such as psoralens. Tans induced by 5-methoxypsoralen (5-MOP) contained in a UVB sunscreen with solar stimulating radiation (SSR) were found to reduce unscheduled DNA synthesis following minimal erythema doses of SSR. Despite the fact that 5-MOP is a weak photocarcinogen, judicious use of 5-MOP-containing sunscreens may offer benefits with regard to protection against various forms of light-induced skin cancer which could outweigh the risk involved. This may be of importance in protecting future generations from the risk of developing malignant melanoma.

### Melanoma in the clinic

To round off the workshop, Dr N. Thatcher (Manchester, UK) gave an overview of current clinical treatments for melanoma. Beginning with the slogan 'If it's small cut it out', he noted that perhaps the major obstruction towards improving the treatment of melanoma is pessimism.

The major current treatments for malignant melanoma are surgery, radiotherapy, adjuvant treatment – so far disappointing and systemic approaches where metastases are present. The Breslow thickness accurately predicts the prognosis – lesions of 0.75 mm or less giving rise to a metastasis rate of 1%, whereas lesions of 3 mm or more are associated with a metastasis rate of 84%. The Breslow thickness would then be a reasonable basis on which to conduct adjuvant therapy after treatment of the primary lesion and before systemic metastases have become apparent. Surgery may also be employed in the treatment of metastatic melanoma of the brain, lung, G.I. tract and superficial lesions in carefully selected patients. Palliative radiotherapy can also be of use, particularly in the treatment of superficial lesions and bone metastases.

The response to systemic therapy (hormones, chemotherapy) is always higher in skin, lymph nodes and favourable sites in soft tissue rather than in visceral organs – liver, bone, etc. Tamoxifen, (trans-2-[4-(1,2-diphenyl-1-butenyl)phenoxy]-N,N-dimethylethylamine), is of great value in breast cancer, and has been used to treat melanoma following the identification in some patients of oestrogen receptors on the melanoma cell. Occasional reports of responses in post-menopausal women have been published although the speaker could not confirm this activity. DTIC(5-(3,3-dimethyl-1-triazino)imidazole-4-carboxamide) has provided the most consistent tumour response rate of about 15%. Other agents in combination regimes also have been tried, including recently cis-platin, with response rates, in some instances, of 40% or more. The use of high dose melphalan (4-[bis(2-chloroethyl)amino]-phenylalanine), DTIC and other agents with autologous bone marrow rescue has not improved the survival although response rate have been increased to above 50% in some studies.

New drugs such as detrorubicin (the glyoxylic acid ester of the anthracycline adriamycin), dibromodulcitol (1,6-dibromo-1,6-dideoxy-D-galactitol), taxol (the tubulin stabilising compound), and the nitrosamino-phosphonic ester fotemustine have shown some activity, particularly the latter even in cerebral metastases.

One important new avenue of treatment involves immunotherapy with interferons and other biological response modifiers such as interleukin-2. These agents also have been used singlularly or in combination with each other, with monoclonal antibodies and cytotoxic agents such as DTIC, cis-platin and flavone acetic acid. Despite the promise of this new treatment, objective tumour responses are still of the order of 20%.

It is clear that the efficacy of current treatments for metastasising melanoma is highly unsatisfactory and that new treatments are urgently needed. Future methods may include hormone therapy, new biological agents, gene therapy and the use of pro-drugs activated by tyrosinase, towards which approach it is hoped this Workshop will have contributed.

As well as the Cancer Research Campaign, the British Association for Cancer Research and the Association for International Cancer Research, the following are thanked for providing financial support: Amgen Ltd., Asta Pharma AG, Beckman Instruments Inc., Boehringer Ingelheim, Bristol-Myers Pharmaceuticals, Ciba Geigy Pharmaceuticals, Farillon Ltd., Institut de Recherches Internationales Servier, Laboratoire Pharmaceutiques Bergaderm S.A., Lilly Industries Ltd., L'Oreal, Sterling Drug Inc., US Air Force Office of Aerospace Research and Development, and Windsor Pharmaceuticals Ltd.

E.J. Land
*CRC Department of Biophysical Chemistry, Paterson Institute for Cancer Research, Christie Hospital and Holt Radium Institute, Manchester M20 9BX, UK*